# Cultural adaptation and validation of the Geriatric Oral Health Assessment Index - GOHAI - Colombian version

**DOI:** 10.25100/cm.v50i2.3999

**Published:** 2019-06-30

**Authors:** Bruno Gutiérrez Quiceno, María Teresa Calzada Gutiérrez, Andrés Fandiño-Losada

**Affiliations:** 1 Universidad del Valle, School of Dentistry, Faculty of Health, Researcher of the Geriatric and Gerontology group. Cali, Colombia.; 2 Universidad del Valle, School of Public Health, Faculty of Health, Researcher CISALVA Institute. Cali, Colombia

**Keywords:** Oral Health, Quality of Life, Validation Studies, Geriatric Dentistry, Geriatric Assessment, Elderly, Psychometrics, dentures, dental care for aged, aged, Salud oral, calidad de vida, estudios de validación, odontología geriátrica, evaluación geriátrica, adulto mayor, psicometría, dentadura, cuidado dental para la vejez, envejecimiento

## Abstract

**Objective:**

This study aims to carry out the cultural adaptation and the validation of the GOHAI scale for the Colombian population.

**Methods:**

Translation process, cultural adaptation, and content and face validity were carried out with a sample of 63 participants as a pretest. The validation counted with a sample of 7,200 subjects, divided into two groups: a work sample (WS) with 3,628 subjects and a confirmatory sample (CS) with 3,572 subjects. Construct, criterion validity and internal consistency were performed for both samples. Test-retest reliability was assessed with a sub-sample of 75 participants

**Results:**

The GOHAI showed an appropriate face and content validity, the pre-test revealed an understandable questionnaire, the scale showed a unidimensional factorial structure and a Cronbach’s Alpha of 0.8. Convergent validity with a self-perception on general health scale pointed to a significant correlation (*p*= 0.0001), while discriminant validity showed significant differences regarding groups according to age group, skin color, educational level, socio-economic level, healthcare affiliation and self-perception about need of dental prostheses. Gender groups did not show significant differences among groups within either sample. The CS showed similar results, differences existed among factorial structures of 2 and 3 factors, and for discriminant validity, the CS showed statistically significant differences for the Area variable not in the WS. Kendall’s test-retest analysis’s correlation is 0.85 (*p*= 0.0000).

**Conclusions:**

The GOHAI scale is valid and reliable enough to be used as a measure of Oral-Health-Related Quality of Life in the Colombian elderly population, also could be applied for other Latin-American populations.

## Resumen

**Objetivo:**

Adaptar culturalmente y validar la escala de autopercepción de salud bucal - Geriatric Oral Health Assessment Index (GOHAI) para la población mayor colombiana.

**Métodos:**

El proceso de traducción, adaptación cultural, contenido y validez aparente se llevaron a cabo en el pre-test con una muestra de 63 participantes. La validación contó con una muestra de 7,200 sujetos, divididos en dos grupos: una muestra de trabajo (WS) con 3,628 sujetos y una muestra confirmatoria (CS) con 3,572 sujetos. Se realizó validez de constructo, criterio y consistencia interna para ambas muestras. La confiabilidad test-re-test se evaluó con una submuestra de 75 participantes.

**Resultados:**

La escala GOHAI mostró condiciones adecuadas de apariencia y contenido, El pre-test mostro un cuestionario entendible y adecuado, la escala arrojo una estructura factorial única y una consistencia interna Alfa de Cronbach de 0,8. La validez convergente con la variable autopercepción en salud general mostró diferencia significativa entre grupos (*p*= 0.0001), la validez discriminante mostro diferencias significativas con las variables grupo de edad, color de piel, nivel educativo, estrato socio-económico, regímenes de salud y autopercepción de necesidad de prótesis dental; la variable Área mostró diferencia significativa en la MC, no en la muestra MT. El análisis test-retest mostro una correlación de Kendall de 0.85 (*p*= 0.0000).

**Conclusión:**

El instrumento GOHAI es válido y confiable y puede ser usado como una medida de Calidad de Vida relacionada con Salud Bucal en personas mayores en Colombia y puede ser aplicado en otras poblaciones de habla hispana de América Latina.

**Remark**

**Table:** 

**1)Why was this study done?**
This study was carried out because it was necessary to validate a scale of oral health related quality of life, GOHAI have been used in several Spanish speaking populations but for the Colombian population was not validated, this process of development would be useful in subsequent research, and also validate the results in terms of quality of oral life of the SABE Health, Welfare and Aging Survey
**2) What did the researchers do and find?**
The researchers carried out a study whit a psychometric strict methodology whit a big sample and a work and confirmatory databases, in order to have a tool to measure oral health related quality of life. It was found that the Colombian Version of the Geriatric Oral Health Assessment Index has appropriate validity and reliability and the researchers of Colombia could use it in future research on that field
**3) What do these findings mean?**
These findings means that Colombia has now an Oral Health Related Quality of Life scale validated for elderly population and also that the results of quality of life in the SABE Survey are completely.

## Introduction

Quality of Life -QL has been defined as people’s individual perception of their position in life within the context of their culture and the systems of values they live with, and with regards to their goals, expectations and concerns. It is a broad concept which is impacted by a person’s physical health, psychological state, degree of independence, social relationships, personal beliefs and his/her relationship with the environment ^1^. It would be worth clarifying that QL is an individual concept, and it may have different meanings according to the field of application ^2^. Oral conditions play an important role, physically and psychologically, in people’s QL, basically interfering in word pronunciation, social life interactions and nutrition. Overall, QL affects the wellbeing and human development as a whole ^3^.

The process of aging creates changes in the social scope, the sensorial perception, and the cognitive and motor functioning of some elderly people (EP) ^4^ ^,^ ^5^. At the oral health level, there are also different characteristics regarding oral tissues and their functions, with teeth loss increasing due to periodontal illness, cavities and injuries to the oral mucosa ^6^ ^,^ ^7^. The lack of teeth and absence of dental prostheses have a direct relationship with health because the inadequate masticatory function produces changes at nutritional level ^8^. Self-realization and self-acceptance are also affected on account of low self-esteem, pain, discomfort and shame before other people during meals or times of socializing. These aspects would affect the quality of life of elders ^9^.

Based on demographic aging and the need of measuring self-perception on elderly people’s oral, multiple measuring scales have been developed through easy-to-approach questionnaires and appropriate predictors of some clinical conditions. These instruments or scales have been validated in several languages. Among the existing instruments, the scales of preference are the Oral Health Impact Proﬁle - OHIP, the Oral Impact on Daily Performances - OIDP, the Geriatric Oral Health Assessment Index - GOHAI, the Subjective Oral Health Status Indicators - SOHSI and the Dental Impact on Daily Living - DIDL ^10^ ^,^ ^11^. 

The GOHAI scale has been employed in Colombia in elderly groups, and the validation process of this instrument has been done in elder population in several countries ^10^ ^,^ ^11^. There are some versions in Spanish from other Latin-American countries, that is why we choose this scale to be applied in the SABE survey, although the validation and adaptation processes have not been recorded thereof ^12^. The Colombian Spanish GOHAI version is considered specific for the Latin-American elderly because previous Latin-American Spanish versions were adapted and validated with institutionalized elderly subjects, and a Spaniard version, which uses colloquial Spanish terms of that country, it is difficult for understanding among Colombians and other Latin-Americans, who have different cultural settings and different manners to express themselves in Spanish. Thus, this study aims to carry out the cultural adaptation and the validation of the GOHAI scale for the Colombian (Spanish speaking) population using a sample of elderly subjects, which also could be applied for other Latin-American Spanish speaking populations.

## Materials and Methods

### Theoretical framework

The validation process was conceptually grounded in the framework exposed by Locker in 1988, which shows different effects on Quality of Life based on changes arising in the oral cavity. This model has been used to elaborate several instruments of oral Quality of Life, as well as previous validations of the GOHAI scale ^13^ ^-^ ^15^.

### Participants SABE Colombia: survey on health, well-being, and aging in Colombia-Study

The study was performed on the subjects participating in the *“Encuesta Salud, Bienestar y Envejecimiento”* (Survey on Health, Well-Being, and Aging in Colombia) *-* SABE Colombia 2015, a national survey which aims to gather information about the aging process among 60 years old and older Colombians. The survey had a field collection time of one year, between 2015-2016. The SABE survey, as well as the GOHAI scale validation study, were endorsed by the Universidad del Valle’s Ethics Committee (Cod. 083/014 and 093/015), and all participants signed an informed consent form. The survey respondents were 23,694 subjects, among them, 4,689 were excluded due to cognitive impairment identified by the Minimental Test. In total, 19,005 elderly people responded to the GOHAI scale. A pre-test of the GOHAI was tested among 63 participants ^16^. 

### Forward- backward translation procedure

The GOHAI scale is a self-reporting instrument made up by 3 dimensions that assess the physical function, the psychosocial function, pain and discomfort. The instrument consists of 12 questions replicated in a Likert scale that confers each answer a score ranging from 1 to 5; the options used by the scale being Always, Often, Sometimes, Seldom and Never. The total score corresponds to the whole sum of each question and deems the oral health as adequate when the score ranks between 57 and 60, moderate between 51 to 56 and low below or equal to 50. The questions corresponding to numerals 1, 2, 4, 6, 8, 9, 10, 11 and 12 exhibit Likert categories from 1 to 5, the others 3, 5 and 7 exhibit the Likert scale inversely ^17^.

The translation process was carried out by two translators external to the whole research process, who met the following criteria: competent in the study languages (English and Spanish), being acquainted or immersed in the culture where the validated scale would be applied and having basic training on health measuring; training understood as having some kind of prior experience on translating instruments or health-issued documents. The objective was to check for changes in the phrasing, semantic and idiomatic equivalency. Upon receiving the translation, an expert committee was formed by a professional on dentistry, doctor on public health, oral rehabilitator, epidemiologist, Master on Gerontology, Master on epidemiology. This committee performed some joint modifications to the initial version, and an adjusted version of the GOHAI instrument was obtained according to experts.

The same translators provided a positive report in the face of the new version, which was the one tested at the pilot test and employed in the fullness of the SABE survey back translated the instrument again.

### Pilot test

It was carried out at three municipalities: Bogotá (Census code: 11001), Ubaté (Census code: 25843) and Soledad (Census code: 8758) to 63 elderly persons, these three were chosen because they are culturally diverse in the country and would help to understand the aspects to be dealt with in different regions. In addition, two are small towns and one the capital city, an important aspect when testing the instrument. 3-5 blocks were selected in each municipality and all the elderly who lived in the blocks were interviewed.

The results showed that the questions were understood by interviewees and interviewers alike. Cronbach’s Alpha was 0.74, reflecting an acceptable internal consistency, on account of which the study was decided to be continued with that GOHAI version. It was decided to not perform factorial analysis at this stage due to an insufficient sample size. 

### Sample

In studies validating a scale, the process that requires a larger sample is the factorial analysis. Some authors define their sample’s size based on the number of items in the scale to be validated; considering between 10 and 20 subjects per item as an appropriate alternative. Other authors recommend a sample size over 500 subjects as good and over 1,000 ones as excellent ^18^ ^,^ ^19^. Thus, the sample for the GOHAI study would have 240 people as it has 12 questions. However, this study took thrice that size because the answers per item showed an asymmetrical distribution, thus considering a total of 720 elderly subjects. Finally, the sample was quintupled in order to enable comparisons among some sub-groups of subjects, as such a sample of 3,600 subjects was planned for both the *working sample* (sample WS) and the *confirmatory sample* (sample CS).

Both samples (WS and CS) were obtained randomly from the 18,863 subjects who responded the GOHAI scale, after eliminating duplicates, atypical data and GOHAI scores below 12 or above 60. Then, two samples (WS and CS), about 3,600 subjects each, were selected using proportional sampling fractions according to the gender, age-groups, and dwelling area (urban vs rural) variables among the 18.863 subjects selected from the whole survey. All the analyses were performed in both samples, in this manner at the end of the study, it counted with a sample of 7,200 subjects, divided into two groups of approximately 3,600 subjects each one.

**Figure 1:**
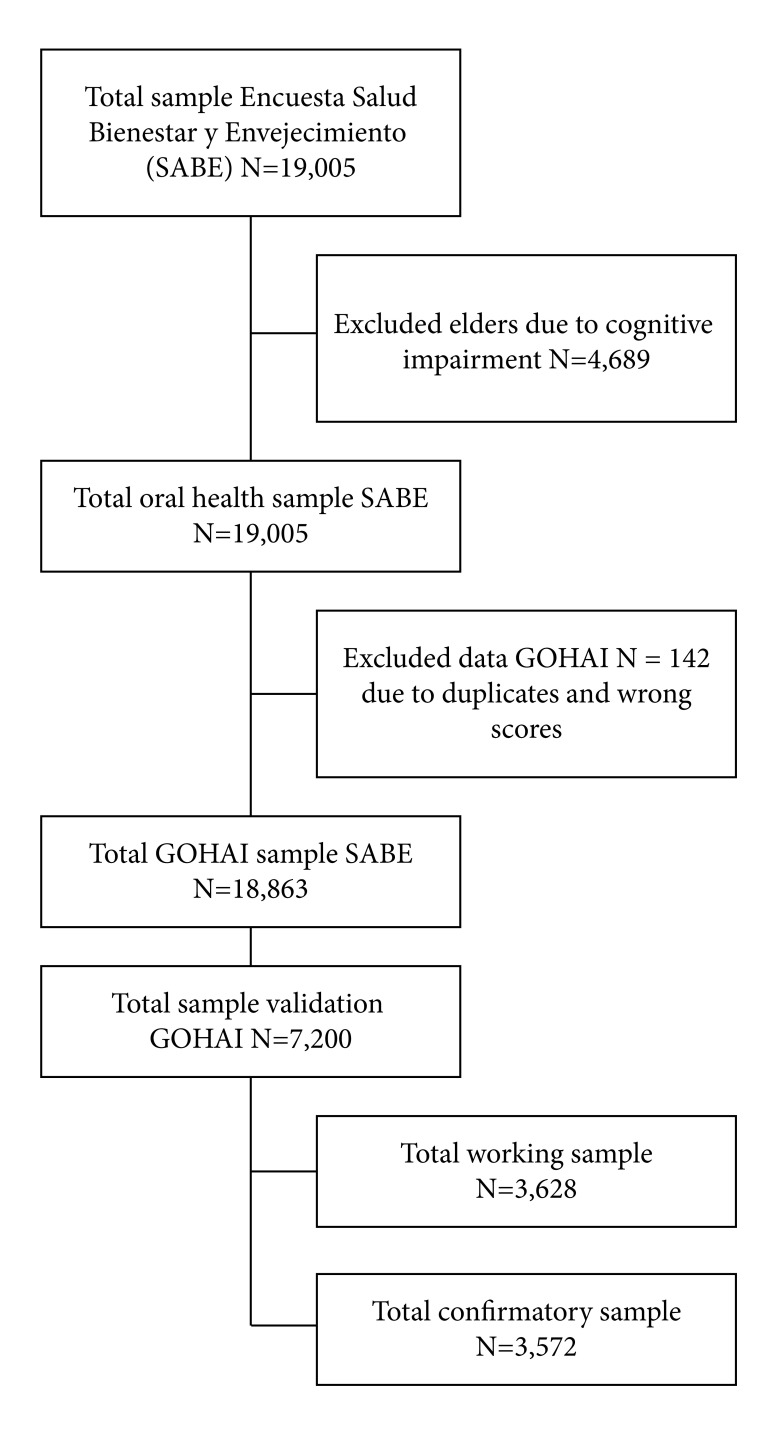
Sampling and validation processes.

The Figure 1 describes the sampling and validation processes.

### Variables

The following existing variables in the SABE Colombia Survey were used: age (60-64, 65-69, 70-74, 75-79 and 80 and above), gender (male-female), educational level (8 sub-groups), dwelling area (rural-urban), socioeconomic level (1-2, 3-4 and 5-6), healthcare affiliation (5 sub-groups), self-perception about dental prostheses need (yes/no), overall oral health self-perception (three sub-groups) and skin color (three sub-groups), with the latter inquired using the pallet of colors from the PEARL in Latin-America project, which displays people’s face pigmentation as a proxy of ethnicity identification ^20^.

### Appearance and content validity

They were appraised through expert analysis by asking themselves if the GOHAI scale truly measures Quality of Life regarding Oral Health, and whether the contents integrate the constructs that would be affected upon the appearance of a favorable or unfavorable oral health condition.

### Statistical analysis

All analyses were carried out on working (WS) and confirmatory (CS) samples. After sampling based on frequency weights, according to the socio-demographic variables already described, work sample consisted of 3.628 registries and confirmatory sample of 3,572; response frequency for both samples and the GOHAI’s translated version are shown in the supplementary Table. *Kruskal-Wallis’* and *Mann-Whitney’s* tests were used to determine if significant differences existed between the samples in relation to the study variables.

### Reliability

Internal consistency was assessed through Cronbach’s Alpha in order to measure homogeneity among items. Using the same interviewees, it was carried out another reliability aspect defined as stability measurement over time, by replicating the GOHAI scale on two different opportunities. 75 subjects were chosen to this part of the validation. The first application was done in the first visit to the elderly whit the application of the SABE survey, the second between 5 and 7 days after the first application. For the test re-test analysis Kendall’s correlation coefficient was used. The coefficient’s interpretation ranges between -1 and +1 pointing to negative or positive associations respectively, while zero (0) means no correlation. 

### Validity

With the purpose of determining the construct’s validity, and stablishing dimensions of variables to be identified, exploratory factorial analysis was used by means of oblique Promax rotation. Factor analysis assumptions were assessed by *Bartlett's* test for sphericity and the *Kaiser-Meyer-Olkin’s* measure of sampling adequacy ^21^ ^,^ ^22^.

Upon being a self-reported scale on a personal construct, which has no gold standard, the GOHAI scale assesses its criteria validity through discriminant and convergence analysis. The discriminant aspect sheds lights on the scale’s ability to differentiate between groups where it must do so, and its inability to differentiate the groups where it must not; this was analyzed though the relationship between the GOHAI total score and socio-demographic variables such as area, age, skin color, educational level, socioeconomic level, healthcare affiliation, self-perception of oral prostheses need and gender. The relationship with the oral health self-perception assessment was used for convergent validity, which inquires how the target construct measured by the studied scale converges to or relates with other scales assessing similar constructs. Kruskal-Wallis’ or Mann-Whitney’s tests were utilized to stablish differences between medium-sized groups depending on the dichotomy or categorical nature of the variables; these tests have been widely used throughout GOHAI validation literature ^2^ ^,^ ^23^.

## Results

Both WS and CS displayed similar characteristics; the Kruskal-Wallis’ and Mann-Whitney’s tests yielded no significant differences (*p* >0.05) for all the variables age, gender, socioeconomic level, marital status, dwelling area, healthcare affiliation, educational level and skin color, that is, the characteristics among the two randomly selected samples were similar.

As a first result, to the experts’ judgement, the GOHAI scale shows adequate content and appearance validity.

### Construct’s validity

Construct analysis for both WS and CS yielded a Kaiser-Meyer-Olkin’s measure above 0.85, which is considered as remarkable, and a significant Bartlett's test (*p* <0.05); both indicating appropriate conditions in order to performing factorial analyses. The factorial structure suggested in both samples was two-factored, considering eigenvalues above 1.0. It is worth clarifying that for both WS and CF a third factor emerged very closely to an eigenvalue of 1.0: 0.96 for WS and 0.98 for CS. Thus, Promax-type rotations were performed for two- and three-factorial structures but the factorial loadings on the GOHAI items were different between both samples. In contrast, the one-factorial structure was consistent over both samples (WS and CS).

### Discriminant validity

Discriminant validity for WS and CS yielded significant differences between the means of variables age, skin color, educational level, socioeconomic level, healthcare affiliation, and self-perception of prostheses need (*p* <0.05). Discriminant differences were not significant for gender (*p* >0.05); for dwelling area was not significant in WS (*p* >0.05), but it was significant in the CS (*p* <0.05). 

### Convergent validity

Convergent validity showed significant results in both WS and CS, between the overall oral health self-perception scale and the total GOHAI score. Table 1 describes results from discriminant and convergent validity.

**Table 1:** Discriminant and Concurrent Validity analysis on working sample (WS) and confirmatory sample (CS).

	Working Sample		Confirmatory Sample	
Variable	Median GOHAI Score (Q1 - Q3***)	*p*	Median GOHAI Score (Q1 - Q3***)	*p*
**Area**				
Urban	54 (48-59)	0.54*	54 (48-58)	0.0018 *
Rural	54 (48-58)		53 (47-57)	
**Age (years)**				
60-64	55 (48-59)	0.01**	54 (48-59)	0.0046 **
65-69	54 (48-59)		55 (48-58)	
70-74	54 (47-58)		54 (48-57)	
75-79	54 (48-58)		54 (48-58)	
80 and over	53 (47-57)		52 (46-56)	
**Skin color**				
Light skin color	55 (48-58)	0.0009 **	54 (48-58)	0.0047 **
Medium skin color	54 (48-58)		54 (48-58)	
Dark skin color	53 (45-57)		53 (47-57)	
**Educational level**				
None	53 (46-57)	0.0001**	52 (46-57)	0.0001 **
Unfinished elementary	53 (47-58)		53 (47-57)	
Finished elementary	55 (48-59)		55 (48.5-58)	
Unfinished high school	55 (49-59)		55 (49-59)	
Finished high school	55 (49-60)		56 (50-59)	
Graduated or ungraduated technician	55 (51-59)		56 (49-59)	
Graduated or ungraduated college	56 (52-60)		56 (52-60)	
Graduated or ungraduated studies	58 (55-59)		57 (53-60)	
**Socio-economical level**				
1-2	54 (47-58)	0.0001**	54 (47-57)	0.0001 **
3-4	55 (49-59)		56 (50-59)	
5-6	56 (52-60)		56 (51-60)	
**Healthcare Affiliation**				
Subsidiary	53 (47-57)	0.0001**	53 (46-57)	0.0000**
Contributive	55 (50-59)		55 (50-59)	
Of exception	53 (47-60)		56 (49-59)	
Special	55.5 (51-60)		55 (50-57)	
Non-affiliate	52 (44-59)		50 (44-57)	
**Prosthesis self-perception need**				
Yes	52 (46-57)	0.0000*	52 (46-57)	0.0000*
No	56 (52-60)		56 (52-60)	
**Gender**				
Male	55 (48-59)	0.14**	54 (47-58)	0.11*
Female	54 (48-58)		54 (48-58)	
**Convergent validity**				
**Overall oral health self-perception**				
Very good/good	55 (49.5-59)	0.0001*	56 (50-59)	0.0001*
Regular	53 (47-57)		53 (46.5-57)	
Bad/Very bad	50 (43-56)		50 (44-56)	

* Mann-Whitney’s test;

** Kruskal-Wallis’s test.

***Q1- Quartile 1 Q3- Quartile 3

### Internal consistency 

Cronbach’s Alpha was 0.80 for both WS and CS, thus demonstrating high correlation and homogeneity among the GOHAI items. The item-scale correlations ranked between 0.49-0.70 for WS and 0.46-0.72 for CS, with an exception for questions 3 and 4 where both samples correlation ranked between 0.35 and 0.4.

### Test-retest reliability

**Figure 2:**
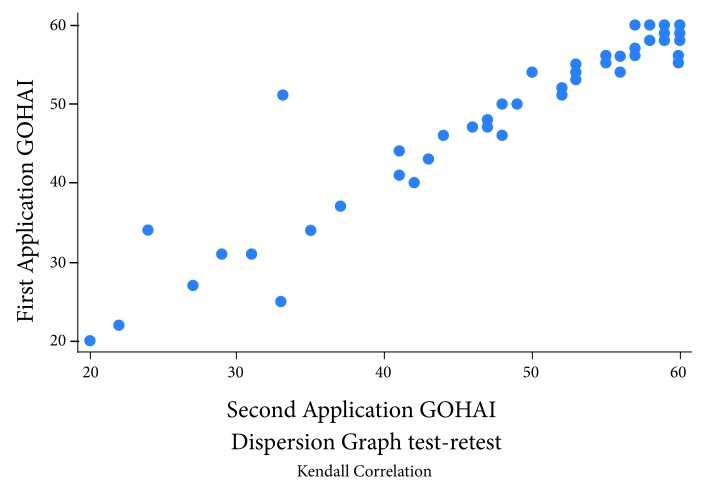
Test- re-test scores scatter graph of the Colombian GOHAI scale.

The results showed a sound correlation between both applications over time; thus, the Kendal tau-B coefficient indicated a significant correlation of 0.85. The scale in both X and Y axis is given in points of the GOHAI score. Figure 2 shows the correlation.

## Discussion

Having a sample of 7,200 elderly subjects participating is appropriate and enabled to carrying out the study tests involving a process of validation, content validity, criteria validity (discriminant and convergent), construct validity and internal consistence on a quite representative Colombian population of 60 years old and more, aside from a reliability test-retest sub-sample sufficient enough to determine the test’s reliability.

Likewise, two sub-samples were used in order to corroborate the validations analyses and results within the SABE Colombia study database.

### Validity of the construct

The construct’s validity based on eigenvalues suggested a two-factorial structure; however, Quality of Life in terms of oral health is shown as a single factor structure (i.e. a dimension). In the original version presenting the GOHAI scale ^10^, only a factor emerged from the factorial analysis and no sub-scales were shown, whereby GOHAI was defined to correspond to a single factor or dimension. The most important difference described through developing this part of the study was the rotation procedure used during the factorial analysis with two and three factors. All previous GOHAI validations used an orthogonal rotation (i.e. Varimax) assuming the phenomena under study to be inwardly independent (non-correlated). Conversely, for the GOHAI Colombian version oblique rotation (i.e. Promax) was used, which allows phenomena, dimensions or factors to be inwardly correlated. The correlation in oral health events is important because we can not separate conditions such as pain or dissatisfaction even more knowing that everything will affect several aspects of the quality of life ^24^. This decision was made taking into account the Oral Quality of Life index’s result, an aspect which depends on other conditions inherent to each person and supported by the theoretical model on which the research was carried out ^15^. 

Regarding the number of factors reported by other studies validating the GOHAI scale, the Spanish version ^25^ found three factors with a small-sized sample of about 100 participants; these results points that the factorial structure of the original study was not replicated ^10^. Likewise, the Greek validation ^26^ also found three factors (or dimensions), using a similar sample size, with three items explained by two factors simultaneously; thus, the authors recommend that structure to be corroborated with a larger sample size. On the contrary, the Mexican validation ^13^ within a much larger sample (n= 695) was able to conclude that the factorial structure corresponded to a single factor, by using the same version of the GOHAI questionnaire as the Spanish validation study. The Portuguese validation ^27^ indicates a factorial structure similar to that found by the Mexican study. The Swiss validation ^28^ suggests a three-factorial structure; nonetheless, after analyzing and verifying the structure the authors concluded that the best way to employ GOHAI is an uni-factorial structure, given the conceptual complexity of assessing oral Quality of Life. The explanation for the factorial structure used by the Swiss study is because the initial and original GOHAI scale was created with the belief that oral health was just a construct which takes dimensions into account within its structure, but no sub-scales ^10^. 

In the current validation study, the structures displayed for scenarios of two and three factors are not consistent with the theoretical structure which underlies the GOHAI scale; verifying that participants do not discriminate between physical, pain-discomfort and psychosocial conceptual constructs, which could be explained by the interrelation of oral Quality of Life’s impact on each of these conceptual dimensions.

The findings above would corroborate the lack of replicability of factorial structure’s of two and three factors between both samples (WS and CS), and supports the choice of a uni-factorial structure for the Colombian GOHAI, hence matching the original scale on a single dimension where Quality of Life within Oral Health is considered a single conceptual construct ^10^.

### Discriminant and convergent validity

The Colombian GOHAI scale allows for discriminating among subjects of different characteristics in terms of age groups, skin color, educational level, socioeconomic level, healthcare affiliation and self-perception of oral prostheses need, which are variables conceptually related with the quality of oral health. In contrast, no discriminant results were found for gender (in both WS and CS samples) neither for dwelling areas in the WS sample.

The discriminant validity results of the Colombian GOHAI scale agree with previous findings in the literature. Differences in GOHAI scores according to age groups have been found in the validation of the French version ^30^, opposite to the Spanish, Greek, Swiss and Chinese versions ^25^ ^,^ ^26^ ^,^ ^28^ ^,^ ^29^, which showed no differences in relation to age; however, this finding was corroborated in both working and confirmatory samples in this study.

Educational level differences were assessed by studies on the Mexican, American and French versions ^13^ ^,^ ^19^ ^,^ ^30^ and showed significant differences, such as the current study has found. In contrast, validation studies of the Greek and Chinese versions did not find educational level differences in GOHAI scores ^26^ ^,^ ^29^. Socioeconomic level and healthcare affiliation are variables assessed differently among countries, which are not properly specified in other validations or not inquired about in most of them.

Furthermore, in the current study, the “colors pallet”, for the assessment of the skin color, was used in discriminant validity analyses. This variable has not been used in other validations. In Colombian settings, skin color is related with discrimination issues and the socioeconomic status ^20^, which could explain the differences of GOHAI scores among different ethnic groups.

The lack of discriminant validity of GOHAI for gender is in line with previous findings from Mexican, American, Spanish, Swiss, Chinese and French validation studies ^13^ ^,^ ^19^ ^,^ ^28^ ^-^ ^30^ which did not report significant differences between men and women in GOHAI scores, according to the quality of oral health conceptualizations.

Dwelling area was previously evaluated only in the French version of the GOHAI ^30^, where it yielded no significant differences; but France is a country with more homogeneous level of development when comparing rural with urban areas. In this manner, more researches are needed on the differences of the GOHAI performance between urban and rural areas in Latin-America, and the role of socioeconomic inequities in such differences.

In relation to the convergent validity, the correlation tests included overall health, oral health self-perception and the self-perception of treatment necessity, showing significant correlations among the constructs.

It is worth clarifying that differences in the results, of the current validation study, are product of transcultural conditions among countries which make these issues incomparable, aside the population’s inherent characteristics and the validation study’s design.

### Internal consistency

In terms of internal consistency, most Cronbach’s alpha values, from previous GOHAI validations, range between 0.8 and 0.9. The Colombian GOHAI attained an internal consistency equal to 0.80, which is consistent with the American, Chinese, Tamil, Persian and Malaysian validations ^19^ ^,^ ^29^ ^,^ ^31^ ^-^ ^33^. The Mexican, Portuguese and Romanian versions ^13^ ^,^ ^27^ ^,^ ^34^ achieved lower internal consistency values, in contrast with the Spanish, Greek, Swiss, French, Dutch, Arab, Japanese and German versions ^25^ ^,^ ^26^ ^,^ ^28^ ^,^ ^30^ ^,^ ^35^ ^-^ ^38^, which report higher internal consistency values.

When withdrawing the GOHAI item 4, the Cronbach’s alpha would come to be 0.81, meaning that internal consistency would change in a single digit, an aspect which does not imply conceptual changes in the internal consistency tests; therefore, the exclusion of item 4 was not accepted.

### Test-Retest Reliability

The authors from the Greek validation ^26^ set the test’s time of re-application one month after it was first applied; the Dutch ^35^ set re-application time between one and two weeks, and the Chinese, Tamil, Arab, German and Chilean validations ^29^ ^-^ ^31^ ^,^ ^36^ ^,^ ^38^ ^,^ ^39^ set the survey’s re-application time to one week after the first application. The Malaysia-validated version ^33^ employed a re-application time between 1 and 14 days; the French took 3 weeks between applications ^30^. As seen in the literature, each author set the time he/she considered appropriate based on his/her experience and previous approaches to the GOHAI scale. In this manner, the expert committee and researchers of the current study defined the re-application time between 5 and 7 days, for the Colombian validation, a period throughout which no changes in Quality of Life are believed to take place on elderly people’s oral health status.

The results as displayed by the GOHAI Colombian version validation, show the correlation measurement to be quite good by yielding a 0.85 Kendall’s coefficient, with a perfect relationship being 1.0, indicating that the Colombian GOHAI has equal or higher test-retest reliability in comparison with the validation of GOHAI versions in other countries; only the Greek, French, Tamil and Dutch versions ^26^ ^,^ ^30^ ^,^ ^31^ ^,^ ^35^ showed slightly higher correlations.

## Conclusions

The Colombian version of the GOHAI scale proved it has appropriate validity and reliability psychometric properties, which suggest this version should be used in longitudinal and cross-sectional research studies about the oral health of the elderly in Colombia. Taking into account the current and the previous validation studies of the GOHAI scale in several Spanish speaking countries (including Spain), it is possible to apply the Colombian version of the GOHAI scale in different Latin-American Spanish speaking countries, adjusting for minor changes in the Spanish wording according to the local vocabulary and other local cultural issues.

The proposed Colombian GOHAI scale will serve, besides, as a public health tool in order to assessing the elderly people’s oral quality of life during the implementation of public health programs or clinical interventions focused on this population. As suggested future studies, it is necessary to perform the confirmatory factorial structure of the GOHAI, aside from assessing the test among different populations, and comparing improvements of the oral quality of life in elderly subjects before and after oral health interventions.

## Supplementary

**Table:** GOHAI response frequencies and translation of items in work sample (WS) and confirmatory sample (CS).

GOHAI Item										
In the past three months…	Always		Often		Sometimes		Seldom		Never	
	WS	CS	WS	CS	WS	CS	WS	CS	WS	CS
How often did you limit the kind or amounts of food you eat because of problems whit your teeth or dentures?	133	140	180	200	171	184	326	344	2,818	2,704
How often did you have trouble biting or chewing any kinds of food, such as firm meat or apples?	449	465	455	444	346	390	444	441	1,924	1,832
How often were you able to swallow comfortably?	169	202	79	97	176	151	379	371	2,825	2,751
How often have your teeth or dentures prevented you from speaking the way you wanted?	651	649	196	176	178	180	302	326	2,301	2,241
How often were you able to eat anything without feeling discomfort?	203	211	203	216	296	307	518	509	2,408	2,329
How often did you limit contacts whit people because of the condition of your teeth or dentures?	125	129	127	140	130	125	322	329	2,924	2,849
How often were you pleased or happy whit the looks of your teeth and gums, or dentures?	495	463	342	354	230	203	496	514	2,065	2,038
How often did you use medication to relieve pain or discomfort from around your mouth.	77	54	86	86	119	133	326	345	3,020	2,954
How often were you worried or concerned about the problems whit your teeth, gums, or dentures?	298	291	246	223	227	236	377	416	2,480	2,406
How often did you feel nervous or self- conscious because of problems whit your teeth, gums or dentures?	224	188	182	199	167	173	346	332	2,709	2,680
How often did you feel uncomfortable eating in front of people because of problems whit your teeth or dentures?	184	188	140	180	165	146	305	291	2,834	2,767
How often were your teeth or gums sensitive to hot, cold or sweets?	200	204	196	214	241	206	362	342	2,629	2,606
