# Supplementary material for: Cultural adaptation and validation of the Geriatric Oral Health Assessment Index - GOHAI - Colombian version
Source: Colomb Med (Cali). 2019 Jun 30;50(2):102–14. doi: 10.25100/cm.v50i2.3999 (PMC6774582; doi:10.25100/cm.v50i2.3999)
Supplement: Translation

# Translation: Adaptación cultural y validación del Geriatric Oral Health Assessment Index - GOHAI - versión Colombia

**Contribución del estudio**

**Table:** 

**1) Por que se hizo este estudio?**
Este estudio se realizó para validar la escala de calidad de vida relacionada con la salud bucal. GOHAI se ha utilizado en varias poblaciones de habla hispana, pero para la población colombiana no ha sido validada. Este proceso de validación será útil en investigaciones posteriores. También validamos los resultados en términos de calidad de vida oral de la Encuesta de Salud, Bienestar y Envejecimiento de SABE
**2) Cuales fueron los resultados mas relevantes?**
Los investigadores hicieron un estudio usando una metodología psicométrica estricta y una gran muestra utilizando bases de datos confirmatorias, para tener una herramienta que nos permita medir la calidad de vida relacionada con la salud oral. Se determinó que la versión colombiana de la escala de autopercepción de salud bucal tiene la validez y confiabilidad apropiadas, por lo que esta versión podrá ser usada en futuras investigaciones en ese campo
**3) Que significan los hallazgos?**
Los investigadores Colombianos podrán disponer de la escala de calidad de vida relacionada con la salud oral validada, para usarse con la población de adultos mayores

## Introducción

La Calidad de Vida (CV) se ha definido como la percepción individual de las personas frente a su posición en la vida, en el contexto de su cultura y los sistemas de valores con los que viven, y con respecto a sus objetivos, expectativas e inquietudes. Es un concepto amplio que se ve afectado por la salud física de una persona, el estado psicológico, el grado de independencia, las relaciones sociales, las creencias personales y su relación con el medio ambiente ^1^. Es necesario aclarar que CV es un concepto individual, y puede tener diferentes significados según el campo de aplicación ^2^. Las condiciones orales juegan un papel importante, física y psicológicamente, en la CV de las personas, básicamente interfiriendo en el habla, las interacciones sociales, las emociones y las expectativas en los tratamientos. En general, la CV afecta el bienestar y el desarrollo humano en su conjunto ^3^. 

El proceso de envejecimiento genera cambios sociales, sobre la percepción sensorial y el funcionamiento cognitivo y motor de algunas personas mayores (PM) ^4^^,^^5^. A nivel de salud bucal, también hay diferentes características con respecto a los tejidos orales y sus funciones, el incremento de la pérdida dental debido a enfermedad periodontal, caries y lesiones en la mucosa oral ^6^^,^^7^. La falta de dientes y la ausencia de prótesis dentales tienen una relación directa con la salud porque la función masticatoria inadecuada produce cambios a nivel nutricional ^8^. La realización y aceptación personal también se ven afectadas debido a la baja autoestima, el dolor, el malestar y la vergüenza ante otras personas durante las comidas o en los momentos de socialización. Estos aspectos pueden afectar la calidad de vida de los ancianos ^9^. 

Sobre la base del envejecimiento demográfico y la necesidad de medir la autopercepción oral de las personas mayores, se han desarrollado múltiples escalas de medición a través de cuestionarios fáciles de abordar y predictores adecuados de algunas condiciones clínicas. Estos instrumentos o escalas han sido validados en varios idiomas. Entre los instrumentos existentes, las escalas de preferencia son el Perfil de Impacto de Salud Oral - OHIP, el Impacto Oral en el Desempeño Diario - OIDP, el Índice de Evaluación de Salud Oral Geriátrica - GOHAI, los Indicadores Subjetivos del Estado de Salud Oral - SOHSI y el Impacto Dental en el Diario Vivir - DIDL^10^^,^^11^.

La escala GOHAI se ha empleado en Colombia en grupos de ancianos, y el proceso de validación de este instrumento se ha realizado en la población de ancianos en varios países ^10^^,^^11^. Existen algunas versiones en español de otros países latinoamericanos, por eso elegimos esta escala para ser aplicada en la encuesta SABE, aunque los procesos de validación y adaptación no se habían registrado en el país ^12^. La versión colombiana de GOHAI en español se considera específica para los adultos mayores latinoamericanos porque las anteriores versiones españolas usadas en latinoamericana se adaptaron y validaron en personas mayores institucionalizadas, y una versión española, que utiliza términos coloquiales en español de ese país, es difícil de entender para la poblacion colombiana e incluso para otros latinoamericanos, que tienen diferentes entornos culturales y diferentes maneras de expresarse en español. Por lo tanto, este estudio tiene como objetivo llevar a cabo la adaptación cultural y la validación de la escala GOHAI para la población colombiana utilizando una muestra de sujetos de edad avanzada, que también podría aplicarse para otras poblaciones hispanohablantes latinoamericanas.

## Materiales y Métodos

### Marco teórico

El proceso de validación se basa conceptualmente en el marco expuesto por Locker en 1988, que muestra diferentes efectos sobre la calidad de vida basado en los cambios que surgen en la cavidad oral. Este modelo se ha utilizado para elaborar varios instrumentos de calidad de vida oral, así como validaciones previas de la escala GOHAI ^13^^-^^15^.

### Participantes SABE Colombia: Encuesta sobre Salud, Bienestar y Envejecimiento en Colombia

El estudio se realizó en los sujetos participantes de la "Encuesta de Salud, Bienestar y Envejecimiento - SABE Colombia 2015”, una encuesta nacional cuyo objetivo era recopilar información sobre el proceso de envejecimiento de colombianos de 60 años y más. La encuesta tuvo un tiempo de recolección de campo de un año, entre 2015-2016. La encuesta SABE, así como el estudio de validación de la escala GOHAI, fueron avalados por el Comité de Ética de la Universidad del Valle (Cod. 083/014 y 093/015), y todos los participantes firmaron el consentimiento informado. Los encuestados fueron 23,694 sujetos, entre ellos, 4,689 fueron excluidos debido al deterioro cognitivo identificado por el filtro establecido con la prueba Minimental AMMSE. En total, 19.005 personas mayores respondieron a la escala GOHAI. Se realizó un pre-test de la escala GOHAI con 63 participantes ^16^.

### Procedimiento de traducción y retro traducción:

La escala GOHAI es un instrumento de autorreporte compuesto por 3 dimensiones que evalúan la función física, la función psicosocial, el dolor y la incomodidad. El instrumento consta de 12 preguntas en una escala Likert que confiere a cada respuesta una puntuación que va de 1 a 5; Las opciones de respuesta son: siempre, casi siempre, con frecuencia, casi nunca y Nunca. 

Las preguntas correspondientes a los números 1, 2, 4 , 6, 8, 9, 10, 11 y 12 exhiben categorías Likert del 1 al 5, las demas preguntas 3, 5 y 7 exhiben la escala Likert inversamente. El puntaje total corresponde a la suma total de cada pregunta y considera que la salud oral es *adecuada* cuando el puntaje se ubica entre 57 y 60, *moderado* entre 51 a 56 y *bajo* por debajo o igual a 50 ^17^.

El proceso de traducción fue realizado por dos traductores externos a todo el proceso de investigación, que cumplieron con los siguientes criterios: competentes en los idiomas de estudio (inglés y español), familiarizados o inmersos en la cultura donde se aplicaría la escala validada y teniendo entrenamiento básico en medición de salud; capacitación entendida como tener algún tipo de experiencia previa en la traducción de instrumentos o documentos emitidos en salud. El objetivo era verificar los cambios en la redacción, la equivalencia semántica e idiomática. Al recibir la traducción, un equipo de expertos compuesto por profesionales en odontología con formación de doctorado en salud pública, especialización en rehabilitación oral, epidemiólogo, magíster en gerontología y en epidemiología realizó algunas modificaciones conjuntas a la versión inicial, y se obtuvo una versión ajustada del instrumento GOHAI según los expertos. Los mismos traductores proporcionaron un informe positivo frente a la nueva versión, que fue la que se probó en la prueba piloto y empleado en la totalidad de la encuesta SABE, intrumento traducido nuevamente por ellos. 

### Prueba piloto

Se llevó a cabo integrando 63 personas mayores en Bogotá (código de censo: 11001), Ubaté (código de censo: 25843) y Soledad (código de censo: 8758). Los tres sitios fueron elegidos por ser culturalmente diversos en el país y ayudaria a comprender los aspectos al tratar diferentes regiones. Además, dos son pueblos pequeños y uno la ciudad capital del pais, un aspecto importante al probar el instrumento. Se seleccionaron 3-5 bloques en cada municipio y se entrevistó a todos los adultos mayores que vivían en los mismos.

Los resultados mostraron que los participantes y los entrevistadores entendieron las preguntas. El Alfa de Cronbach fue de 0.74, lo que refleja una consistencia interna aceptable, por lo que se decidió continuar el estudio con esa versión de GOHAI. No se realizó análisis factoriales en esta etapa debido a un tamaño de muestra insuficiente para ese proceso.

### Muestra

En los estudios de validación de escala, el proceso que requiere una muestra más grande es el análisis factorial. Algunos autores definen el tamaño de su muestra en función del número de preguntas en la escala a validar, considerando entre 10 y 20 sujetos por ítem como una alternativa apropiada. Otros autores recomiendan un tamaño de muestra de más de 500 sujetos como bueno y más de 1,000 como excelente ^18^^,^^19^. Por lo tanto, la muestra para el estudio GOHAI tendría 240 personas, ya que tiene 12 preguntas. Sin embargo, este estudio tomó tres veces ese tamaño porque las respuestas por ítem mostraron una distribución asimétrica, considerando un total de 720 sujetos de edad avanzada. Finalmente, la muestra se quintuplicó para permitir comparaciones entre algunos subgrupos de sujetos, ya que se planificó una muestra de 3,600 sujetos tanto para la muestra de trabajo (WS) como para la muestra confirmatoria (CS).

Después de eliminar duplicados, datos atípicos y puntajes GOHAI por debajo de 12 o por encima de 60, se obtuvieron 18,863 sujetos que respondieron la escala GOHAI en el estudio SABE. Luego, dos muestras (WS y CS) fueron obtenidas al azar, alrededor de 3,600 sujetos cada una, se seleccionaron usando fracciones de muestreo proporcionales de acuerdo con las variables de género, grupos de edad y área de vivienda (urbana - rural) entre los 18,863 sujetos seleccionados de toda la encuesta. Todos los análisis se realizaron en ambas muestras, de esta manera al final del estudio, se contó con una muestra de 7,200 sujetos, divididos en dos grupos de aproximadamente 3,600 sujetos cada uno. La Figura 1 describe los procesos de muestreo y de validación.

**Figura 1:** Proceso de muestreo y validación GOHAI

### Variables

Se utilizaron las siguientes variables existentes en la Encuesta SABE Colombia: edad (60-64, 65-69, 70-74, 75-79 y 80 y más), género (hombre-mujer), nivel educativo (8 subgrupos) , área de vivienda (rural-urbana), estrato socioeconómico (1-2, 3-4 y 5-6), afiliación a la atención médica (5 subgrupos), autopercepción sobre la necesidad de prótesis dentales (sí / no), autopercepción general de salud oral (tres subgrupos) y color de piel (tres subgrupos), esta ultima se preguntó utilizando la paleta de colores del proyecto PEARL en América Latina, que muestra la pigmentación de la cara de las personas como un proxy de identificación étnica ^20^. 

### Apariencia y validez de contenido

Fueron evaluados a través del análisis de expertos acerca de la escala GOHAI y si ella realmente mide la calidad de vida con respecto a la salud bucal, y si los contenidos integran las construcciones que se verían afectadas ante la aparición de una condición de salud bucal favorable o desfavorable

### Análisis estadístico

Todos los análisis se realizaron en las muestras de trabajo (WS) y confirmatorias (CS). Después del muestreo basado en los pesos de frecuencia, de acuerdo con las variables sociodemográficas ya descritas, la muestra de trabajo consistió en 3,628 registros y la muestra confirmatoria de 3,572; La frecuencia de respuesta para ambas muestras y la versión traducida del GOHAI se muestran en la tabla complementaria . Las pruebas de Kruskal-Wallis y Mann-Whitney se utilizaron para determinar si existían diferencias significativas entre las muestras en relación con las variables del estudio.

### Confiabilidad

La consistencia interna se evaluó mediante el Alfa de Cronbach para medir la homogeneidad entre los ítems. Utilizando los mismos entrevistados, se llevó a cabo otro aspecto de confiabilidad definido como medición de estabilidad a lo largo del tiempo, al replicar la escala GOHAI en dos oportunidades diferentes. Se eligieron 75 sujetos para esta parte de la validación. La primera aplicación se realizó en la primera visita a los adultos mayores participantes en la aplicación de la encuesta SABE, la segunda entre 5 y 7 días después de la primera aplicación. Para el análisis de la reevaluación de la prueba se utilizó el coeficiente de correlación de Kendall. La interpretación del coeficiente oscila entre -1 y +1 apuntando a asociaciones negativas o positivas respectivamente, mientras que cero (0) significa que no hay correlación.

### Validez

Con el fin de determinar la validez del constructo y establecer las dimensiones de las variables a identificar, se utilizó un análisis factorial exploratorio mediante rotación oblicua de Promax. Los supuestos del análisis factorial se evaluaron mediante la prueba de esfericidad de Bartlett y la medida de adecuación muestral de Kaiser-Meyer-Olkin ^21^^,^^22^. 

Al ser una escala autoinformada en una construcción personal, que no tiene un estándar de oro, la escala GOHAI evalúa la validez de sus criterios mediante análisis discriminante y de convergencia. El aspecto discriminante arroja la capacidad de la escala para diferenciar entre grupos donde debe hacerlo, y su incapacidad para diferenciar los grupos donde no debe hacerlo; esto se analizó a través de la relación entre el puntaje total de GOHAI y las variables sociodemográficas como área, edad, color de piel, nivel educativo, estrato socioeconómico, afiliación al regimen de salud, autopercepción de la necesidad de prótesis orales y género. La relación con la evaluación de autopercepción de salud oral se utilizó para la validez convergente, que indaga cómo el constructo objetivo medido por la escala estudiada converge o se relaciona con otras escalas que evalúan construcciones similares. Las pruebas de Kruskal-Wallis o Mann-Whitney se utilizaron para establecer diferencias entre los grupos de tamaño mediano según la dicotomía o la naturaleza categórica de las variables; Estas pruebas se han utilizado ampliamente en toda la literatura de validación de GOHAI ^2^^,^^23^. 

## Resultados

Tanto WS como CS mostraron características similares; Las pruebas de Kruskal-Wallis y Mann-Whitney no arrojaron diferencias significativas (*p* >0.05) para todas las variables edad, sexo, estrato socioeconómico, estado civil, área de vivienda, afiliación a la atención médica, nivel educativo y color de la piel, es decir, las características entre las dos muestras seleccionadas al azar fueron similares.

Como primer resultado, a juicio de los expertos, la escala GOHAI muestra una validez de contenido y apariencia adecuada.

### Validez del constructo

El análisis de construcciones para WS y CS arrojó una medida de Kaiser-Meyer-Olkin superior a 0.85, que se considera notable, y una prueba de Bartlett significativa (*p* <0.05); ambos indican condiciones apropiadas para realizar análisis factoriales. La estructura factorial sugerida en ambas muestras fue de dos factores, considerando valores propios superiores a 1.0. Vale la pena aclarar que tanto para WS como para CF surgió un tercer factor muy cercano a un valor propio de 1.0: 0.96 para WS y 0.98 para CS. Por lo tanto, se realizaron rotaciones de tipo Promax para estructuras factoriales de dos y tres, pero las cargas factoriales en los ítems de GOHAI fueron diferentes entre ambas muestras. Por el contrario, la estructura unifactorial fue consistente en ambas muestras (WS y CS).

### Validez discriminante

La validez discriminante para WS y CS arrojó diferencias significativas entre las medias de las variables edad, color de la piel, nivel educativo, nivel socioeconómico, afiliación al regimen de salud y autopercepción de la necesidad de prótesis (*p* <0.05). Las diferencias discriminantes no fueron significativas para el género (*p* >0.05); para el área de vivienda no fue significativa en WS (*p* >0.05), pero fue significativa en el CS (*p* <0.05).

### Validez convergente

La validez convergente mostró resultados significativos tanto en WS como en CS, entre la escala general de autopercepción de salud bucal y la puntuación total de GOHAI. La Tabla 1 describe los resultados de validez discriminante y convergente.

**Tabla 1:** Análisis de la validez discriminante y concurrente en la muestra de trabajo (WS) y en la muestra confirmatoria (CS).

	Muestra de trabajo		Muestra Confirmatoria	
Variable	Mediana GOHAI Score (Q1 - Q3***)	*p*	Mediana GOHAI Score (Q1 - Q3***)	*p*
**Area**				
Urbano	54 (48-59)	0.54*	54 (48-58)	0.0018 *
Rural	54 (48-58)		53 (47-57)	
**Edad (años)**				
60-64	55 (48-59)	0.01**	54 (48-59)	0.0046 **
65-69	54 (48-59)		55 (48-58)	
70-74	54 (47-58)		54 (48-57)	
75-79	54 (48-58)		54 (48-58)	
80 y más	53 (47-57)		52 (46-56)	
**Color de piel**				
Piel Color Claro	55 (48-58)	0.0009 **	54 (48-58)	0.0047 **
Piel Color Medio	54 (48-58)		54 (48-58)	
Piel Color Oscuro	53 (45-57)		53 (47-57)	
**Nivel educativo**				
Ninguno	53 (46-57)	0.0001**	52 (46-57)	0.0001 **
Primaria Incompleta	53 (47-58)		53 (47-57)	
Primaria Completa	55 (48-59)		55 (48.5-58)	
Secundaria Incompleta	55 (49-59)		55 (49-59)	
Secundaria Completa	55 (49-60)		56 (50-59)	
Tecnico completo o incompleto	55 (51-59)		56 (49-59)	
Universitario completo o incompleto	56 (52-60)		56 (52-60)	
Posgrado complete o incompleto	58 (55-59)		57 (53-60)	
**Estrato socio-economico**				
1-2	54 (47-58)	0.0001**	54 (47-57)	0.0001 **
3-4	55 (49-59)		56 (50-59)	
5-6	56 (52-60)		56 (51-60)	
**Regimen de salud**				
Subsidiado	53 (47-57)	0.0001**	53 (46-57)	0.0000**
Contributivo	55 (50-59)		55 (50-59)	
De escepción	53 (47-60)		56 (49-59)	
Especial	55.5 (51-60)		55 (50-57)	
No afiliado	52 (44-59)		50 (44-57)	
**Autopercepcion de necesidad de protesis**				
Si	52 (46-57)	0.0000*	52 (46-57)	0.0000*
No	56 (52-60)		56 (52-60)	
**Genero**				
Masculino	55 (48-59)	0.14**	54 (47-58)	0.11*
Femenino	54 (48-58)		54 (48-58)	
**Validez convergente**				
**Autopercepción feneral de salud oral**				
Muy Buena/Buena	55 (49.5-59)	0.0001*	56 (50-59)	0.0001*
Regular	53 (47-57)		53 (46.5-57)	
Mala/muy mala	50 (43-56)		50 (44-56)	

* Mann-Whitney’s test;

** Kruskal-Wallis’s test.

***Q1- cuartil 1 Q3- cuartil 3

### Consistencia interna

El Alfa de Cronbach fue de 0.80 para WS y CS, lo que demuestra una alta correlación y homogeneidad entre los ítems de GOHAI. Las correlaciones a escala de ítems se ubicaron entre 0.49-0.70 para WS y 0.46-0.72 para CS, con la excepción de las preguntas 3 y 4 donde ambas correlaciones de muestras se ubicaron entre 0.35 y 0.4.

### Fiabilidad Test-retest 

Los resultados mostraron una correlación sólida entre ambas aplicaciones a lo largo del tiempo; así, el coeficiente de Kenu tau-B indicó una correlación significativa de 0,85. La escala en los ejes X e Y se da en puntos de la puntuación GOHAI. La Figura 2 muestra la correlación.

**Figura 2:** Gráfico de dispersión de puntajes test-re-test de la escala GOHAI Colombia.

## Discusión

Contar con una muestra de 7,200 adultos mayores es apropiado y permite llevar a cabo las pruebas de estudio que implican un proceso de validación, validez de contenido, validez de criterios (discriminante y convergente), validez de constructo y consistencia interna en una población colombiana bastante representativa de 60 años y más, aparte de una submuestra de aplicación-re aplicación suficiente para determinar la confiabilidad de la escala.

Asimismo, se utilizaron dos submuestras para corroborar los análisis de validación y los resultados dentro de la base de datos del estudio SABE Colombia.

### Validez de constructo

La validez de constructo basada en valores propios sugirió una estructura de dos factores; sin embargo, la calidad de vida en términos de salud bucal se muestra como una estructura de factor único (es decir, una dimensión). En la versión original que presentaba la escala GOHAI ^10^, solo surgió un factor del análisis factorial y no se mostraron subescalas, por lo que se definió que GOHAI correspondía a un único factor o dimensión. La diferencia más importante descrita a través del desarrollo de esta parte del estudio fue el procedimiento de rotación utilizado durante el análisis factorial con dos y tres factores. Todas las validaciones previas de GOHAI utilizaron una rotación ortogonal (es decir, Varimax) asumiendo que los fenómenos en estudio son internamente independientes (no correlacionados). Por el contrario, para la versión colombiana de GOHAI se utilizó la rotación oblicua (es decir, Promax), lo que permite que los fenómenos, dimensiones o factores se correlacionen internamente. La correlación en los eventos de salud bucal es importante porque no podemos separar condiciones como el dolor o la insatisfacción aún más sabiendo que todo afectará varios aspectos de la calidad de vida ^24^. Esta decisión se tomó teniendo en cuenta que el resultado de calidad de vida oral, es un aspecto que depende de otras condiciones inherentes a cada persona y respaldado por el modelo teórico en el que se realizó la investigación ^15^. 

Con respecto al número de factores informados por otros estudios que validan la escala GOHAI, la versión en español ^25^ encontró tres factores con una muestra de tamaño pequeño de aproximadamente 100 participantes; éstos resultados indican que la estructura factorial del estudio original no fue replicada ^10^. Del mismo modo, la validación griega ^26^ también encontró tres factores (o dimensiones), utilizando un tamaño de muestra similar, con tres elementos explicados por dos factores simultáneamente; por lo tanto, los autores recomiendan que la estructura se corrobore con un tamaño de muestra mayor. Por el contrario, la validación mexicana ^13^ dentro de una muestra mucho más grande (n = 695) pudo concluir que la estructura factorial correspondía a un solo factor, al usar la misma versión del cuestionario GOHAI que el estudio de validación español. La validación portuguesa ^27^ indica una estructura factorial similar a la encontrada por el estudio mexicano. La validación suiza ^28^ sugiere una estructura trifactorial; No obstante, después de analizar y verificar la estructura, los autores concluyeron que la mejor manera de emplear GOHAI es una estructura uni-factorial, dada la complejidad conceptual de la evaluación de la calidad de vida oral. La explicación de la estructura factorial utilizada por el estudio suizo se debe a que la escala GOHAI original se creó con la creencia de que la salud oral era solo un constructo que toma en cuenta las dimensiones dentro de su estructura, pero no subescalas ^10^. 

En el estudio de validación actual, las estructuras mostradas para escenarios de dos y tres factores no son consistentes con la estructura teórica que subyace en la escala GOHAI; corroborando que los participantes no discriminan entre construcciones conceptuales físicas, de incomodidad del dolor y psicosociales, lo que podría explicarse por la interrelación del impacto de la calidad de vida oral en cada una de estas dimensiones conceptuales.

Los hallazgos anteriores corroborarían la falta de replicabilidad de la estructura factorial de dos y tres factores entre ambas muestras (WS y CS), y respaldan la elección de una estructura uni-factorial para el GOHAI colombiano, por lo tanto, coinciden con la escala original en una sola dimensión donde la calidad de vida dentro de la salud oral se considera una construcción conceptual única ^10^. 

### Validez discriminante y convergente

La escala GOHAI colombiana permite discriminar entre sujetos de diferentes características en términos de grupos de edad, color de piel, nivel educativo, estrato socioeconómico, regimen de salud y autopercepción de la necesidad de prótesis orales, que son variables conceptualmente relacionadas con la calidad de la salud bucal. Por el contrario, no se encontraron resultados discriminantes para el género (en las muestras WS y CS) ni para las áreas de vivienda en la muestra WS.

Los resultados de validez discriminante de la escala GOHAI versión Colombia concuerdan con hallazgos previos en la literatura. Se han encontrado diferencias en los puntajes de GOHAI según los grupos de edad en la validación de la versión francesa ^30^, opuesta a las versiones española, griega, suiza y china ^25^^,^^26^^,^^28^^,^^29^ , que no mostraron diferencias en relación con edad; sin embargo, este hallazgo fue corroborado en muestras de trabajo y confirmatorias en este estudio.

Las diferencias en el nivel educativo fueron evaluadas por estudios en las versiones mexicana, estadounidense y francesa ^13^^,^^19^^,^^30^ y mostraron diferencias significativas, como lo ha encontrado el estudio actual. En contraste, los estudios de validación de las versiones griega y china no encontraron diferencias en el nivel educativo en las puntuaciones de GOHAI ^26^^,^^29^ . El nivel socioeconómico y la afiliación a la atención médica son variables evaluadas de manera diferente entre países, que no se especifican adecuadamente en otras validaciones o no se consultan en la mayoría de ellas.

Además, en el estudio actual, la "paleta de colores", para la evaluación del color de la piel, se utilizó en análisis de validez discriminante. Esta variable no se ha utilizado en otras validaciones. En entornos colombianos, el color de la piel está relacionado con problemas de discriminación y el estado socioeconómico ^20^, lo que podría explicar las diferencias de los puntajes de GOHAI entre los diferentes grupos étnicos.

La falta de validez discriminante de GOHAI para el género está en línea con los hallazgos anteriores de estudios de validación mexicanos, estadounidenses, españoles, suizos, chinos y franceses ^13^^,^^19^^,^^28^^-^^30^ que no informaron diferencias significativas entre hombres y mujeres en GOHAI puntajes, de acuerdo con la calidad de las conceptualizaciones de salud bucal.

El área de vivienda se evaluó previamente solo en la versión francesa del GOHAI ^30^ , donde no arrojó diferencias significativas; pero Francia es un país con un nivel de desarrollo más homogéneo al comparar las zonas rurales con las urbanas. De esta manera, se necesitan más investigaciones sobre las diferencias en el desempeño de GOHAI entre las áreas urbanas y rurales en América Latina, y el papel de las inequidades socioeconómicas en tales diferencias

En relación con la validez convergente, las pruebas de correlación incluyeron la salud general, la autopercepción de la salud oral y la autopercepción de la necesidad de tratamiento, mostrando correlaciones significativas entre los constructos.

Vale la pena aclarar que las diferencias en los resultados del estudio de validación actual son producto de condiciones transculturales entre países que hacen que estos problemas sean incomparables, aparte de las características inherentes de la población y el diseño del estudio de validación.

### Consistencia Interna

En términos de consistencia interna, la mayoría de los valores alfa de Cronbach de validaciones anteriores de GOHAI, oscilan entre 0.8 y 0.9. El GOHAI versión Colombia alcanzó una consistencia interna igual a 0,80, que es consistente con las validaciones estadounidense, china, tamil, persa y malasia ^19^^,^^29^^,^^31^^-^^33^ . Las versiones mexicana, portuguesa y rumana ^13^^,^^27^^,^^34^ lograron valores de consistencia interna más bajos, en contraste con las versiones española, griega, suiza, francesa, holandesa, árabe, japonesa y alemana ^25^^,^^26^^,^^28^^,^^30^^,^^35^^-^^38^, que informan valores de consistencia interna más altos.

Al retirar el ítem 4 de GOHAI, el alfa de Cronbach llegaría a ser 0.81, lo que significa que la consistencia interna cambiaría en un solo dígito, un aspecto que no implica cambios conceptuales en las pruebas de consistencia interna; por lo tanto, no se aceptó la exclusión del ítem 4.

### Fiabilidad Test-Retest 

Los autores de la validación griega ^26^ establecieron el tiempo de nueva aplicación de la prueba un mes después de su primera aplicación; los holandeses ^35^ establecieron el tiempo de re-aplicación entre una y dos semanas, y las validaciones china, tamil, árabe, alemana y chilena ^29^^-^^31^^,^^36^^,^^38^^,^^39^ fijaron el tiempo de re-aplicación de la encuesta a una semana después La primera aplicación. La versión validada por Malasia ^33^ empleó un tiempo de nueva aplicación entre 1 y 14 días; los franceses tardaron 3 semanas entre aplicaciones ^30^. Como se ve en la literatura, cada autor establece el tiempo que consideró apropiado en función de su experiencia y enfoques anteriores de la escala GOHAI. De esta manera, el comité de expertos y los investigadores del estudio actual definieron el tiempo de reaplicación entre 5 y 7 días, para la validación colombiana, un período durante el cual no se cree que se produzcan cambios en la calidad de vida en el estado de salud bucal de las personas mayores. 

Los resultados mostrados por la validación de la versión colombiana de GOHAI muestran que la medida de correlación es bastante buena al arrojar un coeficiente de Kendall de 0.85, con una relación perfecta de 1.0, lo que indica que el GOHAI versión Colombia tiene una confiabilidad test - retest igual o mayor en comparación con validación de versiones de GOHAI en otros países; solo las versiones griega, francesa, tamil y holandesa ^26^^,^^30^^,^^31^^,^^35^ mostraron correlaciones ligeramente más altas.

## Conclusiones

La versión colombiana de la escala GOHAI demostró que tiene propiedades psicométricas de validez y confiabilidad apropiadas, lo que sugiere que esta versión podría ser usada en estudios de investigación longitudinales y transversales sobre la salud oral de los adultos mayores en Colombia. Teniendo en cuenta los estudios de validación actuales y anteriores de la escala GOHAI en varios países de habla hispana (incluida España), es posible aplicar la versión colombiana de la escala GOHAI en diferentes países latinoamericanos de habla hispana, ajustando los cambios menores en la redacción española de acuerdo con el vocabulario y otros temas culturales locales.

La escala GOHAI en su versión para Colombia servirá, además, como una herramienta de salud pública para evaluar la calidad de vida oral de las personas mayores durante la implementación de programas de salud pública o intervenciones clínicas enfocadas en esta población. Como se sugiere en futuros estudios, es necesario realizar la estructura factorial confirmatoria del GOHAI, además de evaluar la prueba entre diferentes poblaciones y comparar las mejoras de la calidad de vida oral en adultos mayores antes y después de las intervenciones de salud oral.

## Tabla complementaria

**Tabla:** GOHAI: Frecuencia de respuestas y traducción de los items en las muestras trabajadas (WS) y la muestra confirmatoria (CS)

GOHAI Item										
En los últimos 3 meses, con qué frecuencia…	Siempre		Casi Siempre		Con frecuencia		Casi Nunca		Nunca	
	WS	CS	WS	CS	WS	CS	WS	CS	WS	CS
Tuvo que cambiar la cantidad o la clase de alimentos por problemas con su dentadura o prótesis?	133	140	180	200	171	184	326	344	2,818	2,704
Fue difícil morder o masticar algún alimento porque estaba duro?	449	465	455	444	346	390	444	441	1,924	1,832
Pudo pasar o tragar los alimentos sin problema?	169	202	79	97	176	151	379	371	2,825	2,751
La dentadura o prótesis no lo dejaban hablar bien?	651	649	196	176	178	180	302	326	2,301	2,241
Pudo comer cualquier cosa sin molestias?	203	211	203	216	296	307	518	509	2,408	2,329
Limitó relacionarse o tener contacto con la gente debido al estado de su dentadura o prótesis?	125	129	127	140	130	125	322	329	2,924	2,849
Le gustó cómo se ve su dentadura o prótesis?	495	463	342	354	230	203	496	514	2,065	2,038
Usó medicinas para calmar dolor o molestias en la boca?	77	54	86	86	119	133	326	345	3,020	2,954
Tuvo preocupación por problemas con su dentadura o prótesis?	298	291	246	223	227	236	377	416	2,480	2,406
Se sintió tímido o cohibido por problemas con su dentadura o prótesis?	224	188	182	199	167	173	346	332	2,709	2,680
Le incomodó comer delante de otras personas por problemas con su dentadura o prótesis?	184	188	140	180	165	146	305	291	2,834	2,767
Sintió molestias en su dentadura, al comer o beber cosas calientes, frías o dulces?	200	204	196	214	241	206	362	342	2,629	2,606
